# BMP9‐initiated osteogenic/odontogenic differentiation of mouse tooth germ mesenchymal cells (TGMCS) requires Wnt/β‐catenin signalling activity

**DOI:** 10.1111/jcmm.16293

**Published:** 2021-02-18

**Authors:** Wenping Luo, Linghuan Zhang, Bo Huang, Hongmei Zhang, Yan Zhang, Fugui Zhang, Panpan Liang, Qiuman Chen, Qianyu Cheng, Dongmei Tan, Yi Tan, Jinlin Song, Tianyu Zhao, Rex C. Haydon, Russell R. Reid, Hue H Luu, Michael J. Lee, Mostafa El Dafrawy, Ping Ji, Tong‐Chuan He, Liming Gou

**Affiliations:** ^1^ Chongqing Key Laboratory of Oral Diseases and Biomedical Sciences Stomatological Hospital of Chongqing Medical University Chongqing China; ^2^ Department of Orthopaedic Surgery and Rehabilitation Medicine Molecular Oncology Laboratory The University of Chicago Medical Center Chicago IL USA; ^3^ Department of Respiratory Diseases Stem Cell Biology and Therapy Laboratory Ministry of Education Key Laboratory of Child Development and Disorders The Children's Hospital of Chongqing Medical University Chongqing China; ^4^ Department of Clinical Laboratory Jiangxi Province Key Laboratory of Laboratory Medicine The Second Affiliated Hospital of Nanchang University Nanchang China; ^5^ Chongqing Municipal Key Laboratory of Oral Biomedical Engineering of Higher Education Chongqing China; ^6^ Laboratory Animal Center Chongqing Medical University Chongqing China; ^7^ Department of Surgery Section of Plastic and Reconstructive Surgery The University of Chicago Medical Center Chicago IL USA

**Keywords:** BMP9, canonical Wnt/β‐catenin signalling, osteo/odontogenesis, tooth germ mesenchyme cells, tooth regeneration

## Abstract

Teeth arise from the tooth germ through sequential and reciprocal interactions between immature epithelium and mesenchyme during development. However, the detailed mechanism underlying tooth development from tooth germ mesenchymal cells (TGMCs) remains to be fully understood. Here, we investigate the role of Wnt/β‐catenin signalling in BMP9‐induced osteogenic/odontogenic differentiation of TGMCs. We first established the reversibly immortalized TGMCs (iTGMCs) derived from young mouse mandibular molar tooth germs using a retroviral vector expressing SV40 T antigen flanked with the FRT sites. We demonstrated that BMP9 effectively induced expression of osteogenic markers alkaline phosphatase, collagen A1 and osteocalcin in iTGMCs, as well as in vitro matrix mineralization, which could be remarkably blunted by knocking down β‐catenin expression. In vivo implantation assay revealed that while BMP9‐stimulated iTGMCs induced robust formation of ectopic bone, knocking down β‐catenin expression in iTGMCs remarkably diminished BMP9‐initiated osteogenic/odontogenic differentiation potential of these cells. Taken together, these discoveries strongly demonstrate that reversibly immortalized iTGMCs retained osteogenic/odontogenic ability upon BMP9 stimulation, but this process required the participation of canonical Wnt signalling both in vitro and in vivo. Therefore, BMP9 has a potential to be applied as an efficacious bio‐factor in osteo/odontogenic regeneration and tooth engineering. Furthermore, the iTGMCs may serve as an important resource for translational studies in tooth tissue engineering.

## 1 INTRODUCTION

Tooth is a highly mineralized organ arising from the tooth germ, which is induced by sequential and reciprocal interactions between the immature epithelium and the underlying cranial neural‐crest‐derived mesenchyme in the developing embryo.^1^ Most of the dental tissues are formed by dental ectomesenchyme, which is formed by condensed cells originated from cranial neural‐crest‐cells during embryonic development. Following tooth germ formation, cells in tooth germ differentiate into multiple lineages of cells forming tooth tissues, including ameloblast, odontoblast, pulp cells and periodontal ligament cells. These cells secrete extracellular matrix proteins for mineral deposition and then form hard tissues of tooth, such as enamel, dentin and cementum.^2^ Tooth germ mesenchyme determines both tooth identity and the ability to reprogram nondental epithelium to tooth fate, as a result of transmission of the odontogenic potential from epithelium to the mesenchyme in mouse tooth between embryonic days 11 and 12, which was demonstrated by the classic tissue recombination studies.^3^, ^4^, ^5^

While regeneration of fully functional teeth utilizing autologous bioengineered tooth germ transplantation^6^ may provide great provision for improving clinical outcomes in cases involved with tooth loss, harnessing the natural regenerative potential of cranial neural‐crest‐derived mesenchyme cells in tooth‐forming tissue may provide an alternative solution to reconstruct defects of tooth hard tissue and maintain pulp vitality.^7^ For this reason, the stem cells discovered in adult tooth, including the mesenchymal stem cells in dental papilla, pulp and periodontal ligament have attracted intensive attention during the past decades for they are capable of differentiating into cells forming dentin/cementum and periodontal ligament. However, it is unclear whether tooth germ mesenchymal cells (TGMCs) possess osteo/odontoblastic differentiation potential.

Numerous signalling pathways have been implicated in regulating the fate and lineage determination of tooth mesenchyme, including Wnt/β‐catenin pathway and BMP signalling.^8^, ^9^ Nonetheless, the detailed mechanism underlying tooth development from TGMCs remains to be fully understood. We have demonstrated in a previous study that BMP9 (aka, growth and differentiation factor 2, or GDF2) possesses great potential to induce osteogenic, adipogenic and, to a lesser extent, chondrogenic differentiation of mesenchymal stem cells (MSCs),^10^, ^11^ and that BMP9 and canonical Wnt signalling act synergistically to induce osteo/odontoblastic differentiation of stem cells harvested from apical papilla of mature lower incisor teeth, which is considered as a subpopulation of MSC‐like cells.^12^, ^13^

In this study, we investigated the role of Wnt/β‐catenin signalling in BMP9‐induced osteogenic/odontogenic differentiation of TGMCs. To overcome the technical challenge of maintaining primary dental germ cells, we first established the reversibly immortalized TGMCs (iTGMCs) derived from young mouse mandibular molar tooth germs. We demonstrated that BMP9 effectively induced the expression of osteogenic markers and in vitro matrix mineralization, which could be effectively blunted by β‐catenin knockdown. In vivo implantation of BMP9‐stimulated iTGMCs induced robust formation of ectopic bone, knocking down β‐catenin expression in iTGMCs significantly diminished BMP9‐induced ectopic bone formation. Taken together, these discoveries demonstrate the osteogenic/odontogenic ability of engineered iTGMCs upon BMP9 stimulation, but this process requires the participation of canonical Wnt signalling. Therefore, BMP9 has the potential to be explored as an efficacious bio‐factor for osteo/odontogenic tissue engineering and tooth engineering.

## 2 MATERIALS AND METHODS

### 2.1 Cell culture

The HEK‐293 cell line (ATCC) was maintained in complete Dulbecco's Modified Eagle's Medium (DMEM) containing 10% foetal bovine serum (FBS, Invitrogen), 100 units of penicillin and 100 mg of streptomycin at 37°C in 5% CO_2_. The recently engineered 293pTP line was used for adenovirus amplification.^14^ Both cell lines were maintained in complete DMEM. Unless indicated otherwise, all chemicals were purchased from Sigma‐Aldrich or Fisher Scientific.

### 2.2 Isolation and immortalization of mouse TGMCs from late bell stage molar tooth germs

All animal studies were conducted following the guidelines approved by Institutional Animal Care and Use Committee (IACUC). Mandibles were isolated from euthanized newborn male CD1 mice. Mandibular molar tooth germs were retrieved from the surrounding tissue, rinsed by sterile phosphate‐buffered saline (PBS), and kept in 1.2 U/ml of DispaseII and incubated at 37°C for 40 minutes. After being washed in plain DMEM medium, epithelium and mesenchyme of the tooth germs were separated with fine needles. The Mesenchymal tissues were treated once with 0.25% trypsin (Sigma), 50 U/mL collagenase I and 20 U/mL DNase I for 10 minutes at 37°C; twice with 100 U/mL collagenase I (Worthington) for 10 minutes at 37°C; and once with 0.25% trypsin and 20 U/mL DNase I for 5 minutes at 37°C. The dissociated cells were plated in 6‐well cell culture plates after washed in complete DMEM, and then incubated for 24 hours at 37°C. Adherent cells were used as TGMCs. Aliquots were kept in a liquid nitrogen tank. All TGMCs used in this study were within 5 passages.

### 2.3 Establishment of reversibly immortalized tooth germ mesenchymal cells (iTGMCs)

The use of the retroviral vector SSR #41 to express SV40 T antigen flanked with the FRT sites have been described previously.^15^, ^16^, ^17^, ^18^ Briefly, the SSR #41 vector and pCL‐Ampho packaging vector were co‐transfected into293 Phoenix Ampho (293PA) cells to produce the packaged retrovirus. Exponentially growing TGMCs were infected with the SSR #41 retrovirus and subjected to hygromycin B selection (0.4mg/mL) for 3‐5 days twice in complete DMEM at 37°C, yielding the stably immortalized tooth germ mesenchymal cells, designated as the iTGMC line.

### 2.4 Generation and amplification of recombinant adenoviruses expressing BMP9, Wnt3A, Flippase (FLP) and GFP

AdEasy technology was used to generate recombinant adenoviruses as described previously.^19^ Briefly, the coding regions of human BMP9, mouse Wnt3A and FLP recombinase were cloned into an adenoviral shuttle vector, respectively, then these vectors were used to generate recombinant adenoviruses in HEK‐293 or 293pTP cells. The resulting adenoviruses were designated as Ad‐BMP9, Ad‐Wnt3A and Ad‐FLP, both of which also express GFP to monitor infection efficiency. Analogous adenovirus expressing only GFP (Ad‐GFP) was used as control. Polybrene (4‐8 mg/mL) was added to enhance infection efficiency as previously reported.^20^

### 2.5 Construction of multiplex siRNA expression system targeting mouse β‐catenin and the generation of stable iTGMC‐KD and iTGMC‐Ctrl lines

The single vector‐based multiplex expression of siRNAs targeting the coding regions of mouse β‐catenin (NM_007614.3) were constructed by using the BSG Versatile shotgun cloning strategy as recently reported.^21^ Briefly, the 3 siRNA sites targeting β‐catenin were driven by the opposing U6 and H1 promoters into pSEB361‐BSG destination vectors, yielding pBSG361‐simBC. Vector containing the scrambled sites which do not target any significant known human and rodent transcripts was also constructed as a control (eg, pBSG361‐siControl). To generate retrovirus supernatants for infecting subconfluent iTGMCs, the resultant retroviral transfer vectors pSEB361‐simBC and pSEB361‐siControl were co‐transfected with retroviral packaging plasmids into the293PA cells as described in a previous study.^18^ Cells were subjected to blasticidin S selection (final concentration at 3 µg/mL) for 5 days after 36‐48 hours post viral infection. The resultant stable lines were designated as iTGMC‐KD, and iTGMC‐Ctrl, respectively.

### 2.6 RNA isolation and quantitative RT‐PCR (qPCR)

Total RNA was isolated by using TRIZOL Reagents (Invitrogen), and cDNA templates were generated by reverse transcription reactions with hexamer and MMuLV reverse transcriptase (New England Biolabs). QPCR was carried out as described.^22^ The primers used in this study are show in Table 1. SYBR Green‐based qPCR analysis was carried out by using the thermo cycler CXF‐Connect (Bio‐Rad) as described elsewhere.^23^, ^24^, ^25^, ^26^ All qPCR reactions were done in triplicate. All samples were normalized by the expression level of GAPDH.

**TABLE 1:** List of primers used for qPCR analysis

Primer name	Forward	Reverse
*GAPDH* Mus	5′‐CTTTGTACAGCGGCCTCTTC‐3′	3′‐CAGGACATGACACCCAAGTG‐5′
*SV40 T* Mus	5′‐GGTGGGTTAAAGGAGCATGA‐3′	3′‐TAGTGGCTGGGCTGTTCTTT‐5′
*CTNNB1* Mus	5′‐GAGCCTGCCATCTGTGCT‐3′	3′‐ TGGTGGGTGCAGGAGTTT‐5′
*Axin2* Mus	5′‐ CTCCCCACCTTGAATGAAGA −3′	3′‐ ACTGGGTCGCTTCTCTTGAA‐5′
*C‐myc* Mus	5′‐ AGTGCATTGACCCCTCAGTG −3′	3′‐GTGTCTCCTCATGCAGCACT‐5′
*Col1a1* Mus	5′‐GAGCGGAGAGTACTGGATCG‐3′	3’‐GCTTCTTTTCCTTGGGGTTC‐5′
*Osteocalcin* (OCN) Mus	5′‐ CCTTCATGTCCAAGCAGGA −3′	3′‐ GGCGGTCTTCAAGCCATAC −5’

### 2.7 Immunofluorescence staining

Immunofluorescence staining was performed as previously described.^10^ Briefly, cells were first infected with Ad‐Wnt3A or Ad‐GFP for 48 hours or directly fixed with methanol, permeabilized with 1% NP‐40, and blocked with 10% BSA, followed by incubating with primary antibodies. Concentrations of primary antibodies used are shown in Table 2. Of 2 µg/mL Donkey Anti‐Rabbit IgG Secondary Antibody labelled with Alexa Fluor 594 (A‐21207, Jackson ImmunoResearch Laboratories) or 2 µg/mL Donkey Anti‐Rabbit IgG Secondary Antibody labelled with Alexa Fluor 488 ([A‐21206, Thermo Scientific]) were used to mark the targeted cells. Stains without primary antibodies, or with control IgG, were used as negative controls.

**TABLE 2:** List of primary antibodies used for immunofluorescence staining

Antibodies	Concentration	Cat. No. and Manufacture
CD166	200 µg/mL	bs‐1251R, Bioss
CD146	200 µg/mL	bs‐20677R, Bioss
CD44	200 µg/mL	bs‐4916R, Bioss
C‐kit	200 µg/mL	bs‐10005R, Bioss
Vimentin	1 µg/mL	sc‐6260, Santa Cruz Biotechnology
Beta‐catenin	1 µg/mL	sc‐7963, Santa Cruz Biotechnology
BMP2	1 µg/mL	sc‐137087, Santa Cruz Biotechnology
BMP6	1 µg/mL	sc‐57042, Santa Cruz Biotechnology
BMP7	1 µg/mL	sc‐53917, Santa Cruz Biotechnology
BMP9	1 µg/mL	sc‐514211, Santa Cruz Biotechnology
BMPRII	1 µg/mL	sc‐393304, Santa Cruz Biotechnology
OPG	1 µg/mL	sc‐390518, Santa Cruz Biotechnology
Smad1/5/8	1 µg/mL	sc‐6031‐R, Santa Cruz Biotechnology

### 2.8 Alkaline phosphatase (ALP) activity assay

Alkaline phosphatase activity was assessed using the Great ESCAPEe SEAP Chemiluminescence assay kit (BD Clontech) and histochemical staining assay (using a mixture of 0.1 mg/mL napthol AS‐MX phosphate and 0.6 mg/mL Fast Blue BB salt) as described.^27^ Each assay condition was performed in triplicate and the results were repeated in at least 3 independent experiments. ALP activity was normalized by total cellular protein concentrations among the samples.

### 2.9 Matrix mineralization assay (Alizarin Red S staining)

The iTGMCs were seeded in 24‐well cell culture plates and infected with Ad‐BMP9 or Ad‐GFP. Infected cells were cultured in the presence of ascorbic acid (50 mg/mL) and β‐glycerophosphate (10 mM). Mineralized matrix nodules were stained by Alizarin Red S staining 14 days after infection as described.^28^ Briefly, cells were fixed with 2.5% (v/v) glutaraldehyde at room temperature for 10 minutes and washed with PBS before incubated with 2% Alizarin Red S (Sigma‐Aldrich) for 20 minutes. Excess dye was washed off by PBS after incubation. The staining of calcium mineral deposits was recorded under bright‐field microscopy.

### 2.10 Oil Red O staining assay

The iTGMCs were seeded in 24‐well cell culture plates, infected with Ad‐BMP9 or Ad‐GFP, and then cultured for 10 days before Oil Red O staining.^10^ Then cells were fixed with 10% formalin at room temperature for 20 minutes. After washed with PBS, the fixed cells were stained with freshly prepared Oil Red O solution (6 parts saturated Oil Red O dye in isopropanol plus four parts water) at 37°C for 30‐60 minutes, followed by washing with 70% ethanol and distilled water.

### 2.11 Subcutaneous iTGMCs cell implantation, ectopic bone formation and micro‐computed tomography (μCT) analysis

All animal studies were conducted under the guidelines approved by the Institutional Animal Care and Use Committee (IACUC). The thermo‐responsive PPCNg was used as a scaffold for the delivery of BMP9‐stimulated iTGMCs for bone formation as described.^29^ The iTGMCs‐mediated ectopic bone formation was carried out as previously described.^19^ Briefly, iTGMC‐Ctrl and iTGMC‐KD were infected with Ad‐BMP9 or Ad‐GFP for 20 hours, collected and resuspended in 80 μL PPCNg mix (40 μL PPCN + 40 μL 0.2% gelatine), or cells only (80 μL PBS). The infected iTGMC‐Ctrl or iTGMC‐KD + PPCNg mixture were kept on ice and injected subcutaneously into the flanks of athymic nude (nu/nu) mice (Beijing HFK Bioscience, 6‐8‐week‐old, male, 10^6^ cells per injection, 4 sites per mouse, n = 5/group). Animals were sacrificed 4 weeks after implantation. The implantation sites were retrieved for 15μ micro‐CT imaging. All specimens were imaged using the vivaCT 40 preclinical imaging system (Scanco Medical AG) and all image data and 3D volumetric data were analysed using μCT V6.1 software.^13^

### 2.12 Histological evaluation and Trichrome staining

Retrieved tissues were fixed, decalcified in 10% buffered formalin, then embedded in paraffin. Serial sections of the embedded tissue blocks were stained with haematoxylin and eosin (H & E) and Trichrome staining as previously described.^27^

### 2.13 Tumorigenicity of iTGMCs and Xenogen bioluminescence imaging

The tumorigenic potential of iTGMCs was assessed by Xenogen bioluminescence imaging and compared with the human oral cancer line SCC‐15. SCC‐15 and iTGMC cells labelled with firefly luciferase^15^, ^16^, ^17^, ^18^ were designated as SCC‐15‐FLuc and iTGMC‐FLuc, respectively. Exponentially growing SCC‐15‐FLuc and iTGMC‐FLuc cells were collected and injected subcutaneously into the flanks of athymic nude mice (Beijing HFK Bioscience, 6‐8‐week‐old, male, 10^6^ cells per injection, 4 sites per mouse, n = 5/group). Injected animals were subjected to bioluminescence imaging after 2 days and 14 days after implantation using Xenogen IVIS 200 imaging system.^30^, ^31^, ^32^ Quantitative analysis was conducted with Xenogen's Living Image software.^32^

### 2.14 Statistical analysis

All quantitative assays were performed in triplicate and/or repeated 3 times. Analysis results were expressed as mean ± SD. Statistical significances were determined by one‐way analysis of variance or the student's *t* test. A value of *P* < .05 was considered statistically significant.

## 3 RESULTS

### 3.1 The SV40 T antigen‐immortalized TGMCs (iTGMCs) exhibit long‐term proliferative activity in vitro

**FIGURE 1:**
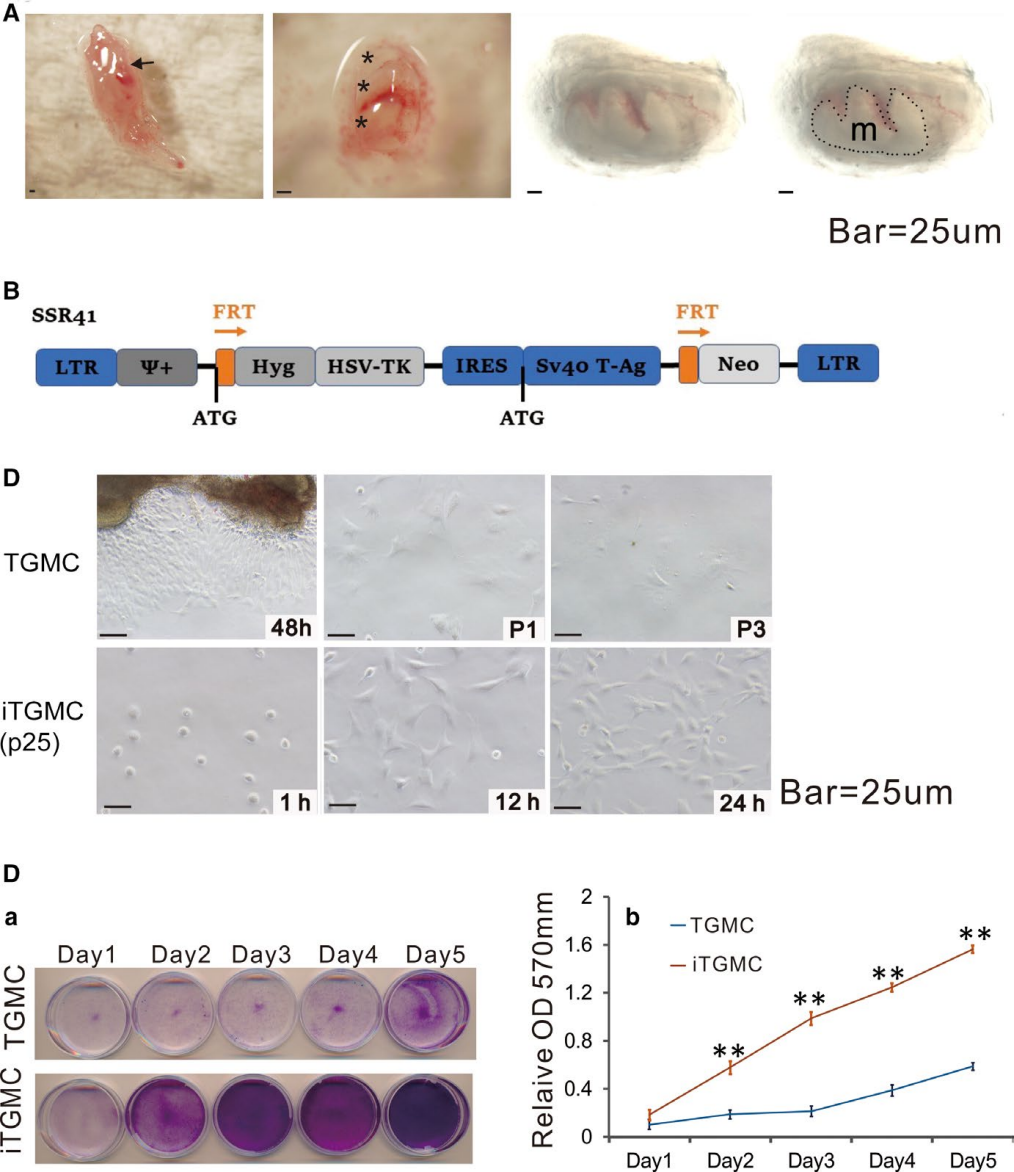
Isolation of mouse tooth germ mesenchyme cells (TGMCs) from mandibular late bell stage molar tooth germs and reversible immortalization of TGMCs (iTGMCs). A, Isolation of late bell stage molar tooth germs from mandible on postnatal day 0. (Short arrow, mandibular molar tooth germs attached to the mandible. *, dissected mandibular molar tooth germs. m, tooth germ mesenchyme (Captured under transmission light). B, Schematic representation of immortalization vector SSR#41, which contains the hygromycin and SV40 T antigen expression cassette flanked with FRT sites and can be removed by the Flippase (FLP) recombinase. C, Morphology of the primary TGMCs and iTGMCs. Bar = 25µM. (a) TGMCs were harvested from digested tooth germs mesenchyme after 48h culture and maintained up to three passages(P3). (b) iTGMCs were seeded at a low cell density and photographed at the indicated time point. iTGMCs were readily maintained indefinitely (passage 25 is shown). D, Cell proliferation assessed by crystal violet staining. (a) TGMCs (P2) and iTGMCs were seeded at 30 mm dish with the same initial density and then fixed with paraformaldehyde for crystal violet staining after cultured for the indicated time scale. (b) The stained cells were dissolved in 10% acetic acid and optical absorbance was measured at 570 to 590 nm. (**, *P* < .001)

To establish reversibly immortalized mouse TGMCs that retain long‐term proliferation capability, tooth germ mesenchyme was dissected from the mouse mandibular molar teeth, and the TGMCs were isolated and cultured (Figure 1A). The adherent cells were infected with retroviral SSR#41 vector that expresses SV40 T antigen and selection marker hygromycin (Figure 1B). After hygromycin selection for 3 days, the surviving cells were replated. The resultant pooled immortalized TGMCs were designated as iTGMCs (Figure 1C).

The proliferative activities were compared between the iTGMCs and the primary TGMCs. We found that when seeded at a similar initial cell density, the iTGMCs reached confluence faster than the primary TGMCs as demonstrated by crystal violet staining assay (Figure 1D‐a). Quantitative assessment indicated that iTGMCs grown significantly higher numbers of cells at each time point than that of TGMCs (Figure 1D‐b). Taken together, these results demonstrate that the iTGMCs can be stably maintained in culture condition and exhibit a high proliferative rate.

### 3.2 FLP recombinase can reverse SV40 T antigen‐mediated immortalization of iTGMCs which also express mesenchymal markers

**FIGURE 2:**
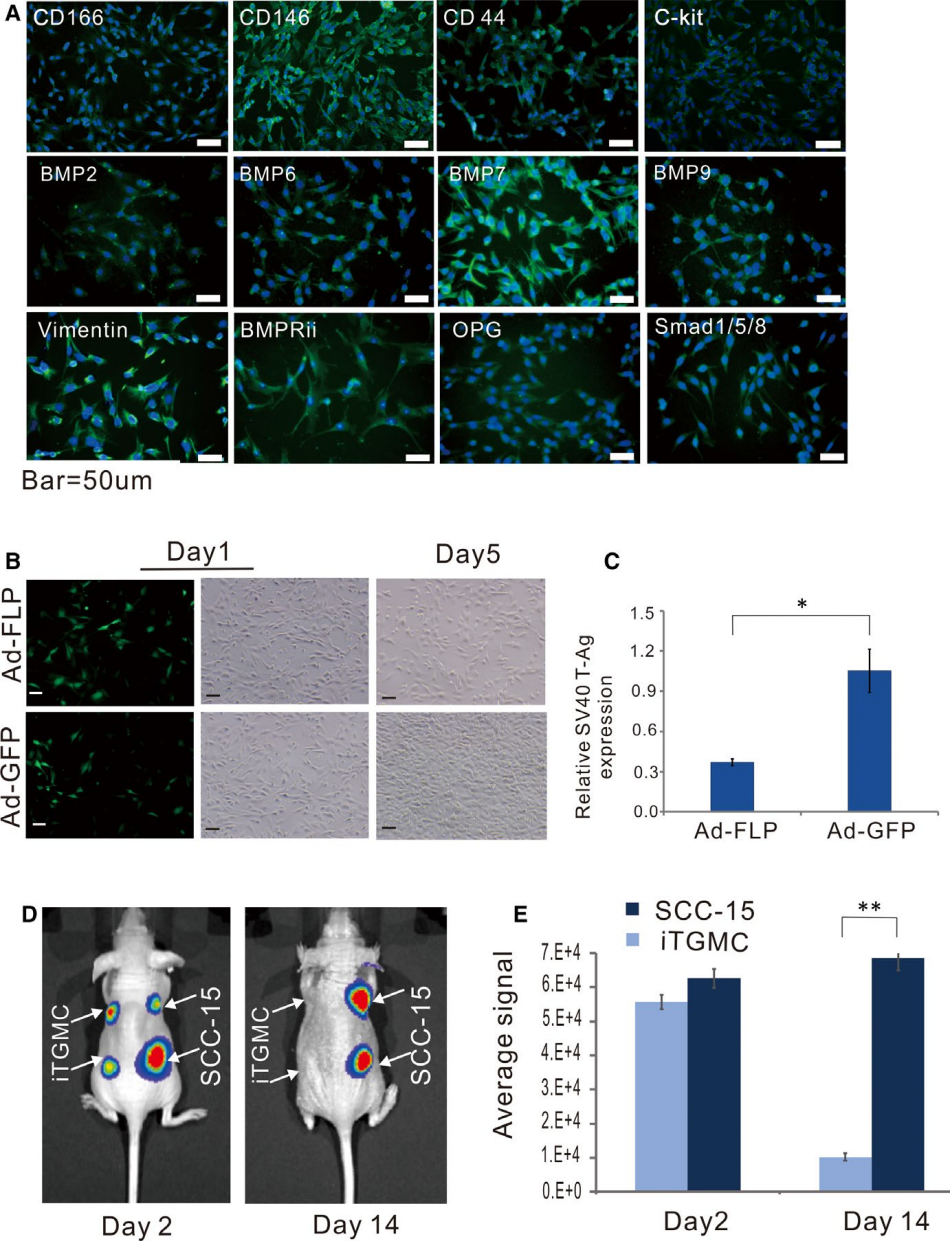
Characterization of reversibly immortalized TGMCs (iTGMCs). A, The proliferative activity of iTGMCs decreases after removal of SV40 T antigen via FLP recombinase. B, Removal of SV40 T antigen in iTGMCs confirmed by RT‐qPCR analysis 3 days after infection. (^*^ *P* < .05). C, Expression of mesenchymal and/or progenitor markers in iTGMCs. IgG and minus primary antibody staining were performed as negative controls (not shown). D, Analysis of the tumorigenic risk in vivo. SCC‐15 and iTGMCs stably expressing the firefly luciferase were injected into the athymic nude mice subcutaneously. Compared with the SCC‐15 group, no masses were detectable in the iTGMCs group at day 14. E, The average bioluminescence signals were quantitatively analysed by the Living Image software. (**P < .01). Representative results are presented

Immunofluorescence staining was used to assess whether the established iTGMCs expressed the biomarkers of mesenchymal progenitors. We found that Vimentin, C‐kit, CD44, CD146, CD166, was strongly expressed in the cytoplasm, and the expression of BMP2, BMP6, BMP7, BMP9, OPG and Smad1/5/8 was also detectable (Figure 2A). Thus, these results demonstrated the iTGMCs exhibited the mesenchymal characteristics.

To test whether the SV40 T antigen‐mediated immortalization could be effectively reversed in iTGMCs, a recombinant adenoviral vector Ad‐FLP was constructed as an effective tool to deliver FLP into iTGMCs.^33^, ^34^ We found that the Ad‐FLP infected iTGMCs grew at a significantly decreased rate (Figure 2B). The efficient removal of SV40 T antigen by FLP was further confirmed by qPCR analysis in the Ad‐FLP infected iTGMCs (Figure 2C). Collectively, these results convincingly indicate that the immortalization phenotype of the iTGMC could be effectively reversed by FLP recombinase and the proliferative activity be diminished accordingly.

### 3.3 The iTGMCs are not tumorigenic in xenograft cell implantation assay in athymic nude mice

As SV40 T antigen is capable of stimulating cell proliferation,^14^ we tested the tumorigenic potential of the established iTGMCs in comparison with a human oral cancer line SCC‐15. SCC‐15‐FLuc and iTGMC‐FLuc cell lines were established and injected into the athymic nude mice subcutaneously, respectively. The animals were subjected to whole body live bioluminescence imaging at different time points after implantation. While similar bioluminescence signals ware readily detectable in all injected animals in both groups on day two after injection, the signals decreased rather to an undetectable level in the iTGMCs‐injected mice on day 14 (Figure 2D), this result was further confirmed by a quantitative analysis (Figure 2E). We monitored the animals for up to four weeks, no tumours or masses were observed in the injection sites or other sites in the iTGMC‐FLuc injection group, while the SCC‐15‐FLuc injection group formed readily detectable tumour masses (data not shown). Collectively, these results strongly indicate that the iTGMCs may be non‐tumorigenic in vivo even though they possess long‐term proliferative activity in vitro.

### 3.4 BMP9 effectively induces osteogenic/odontogenic activity of iTGMCs, which requires the participation of canonical Wnt signalling

**FIGURE 3:**
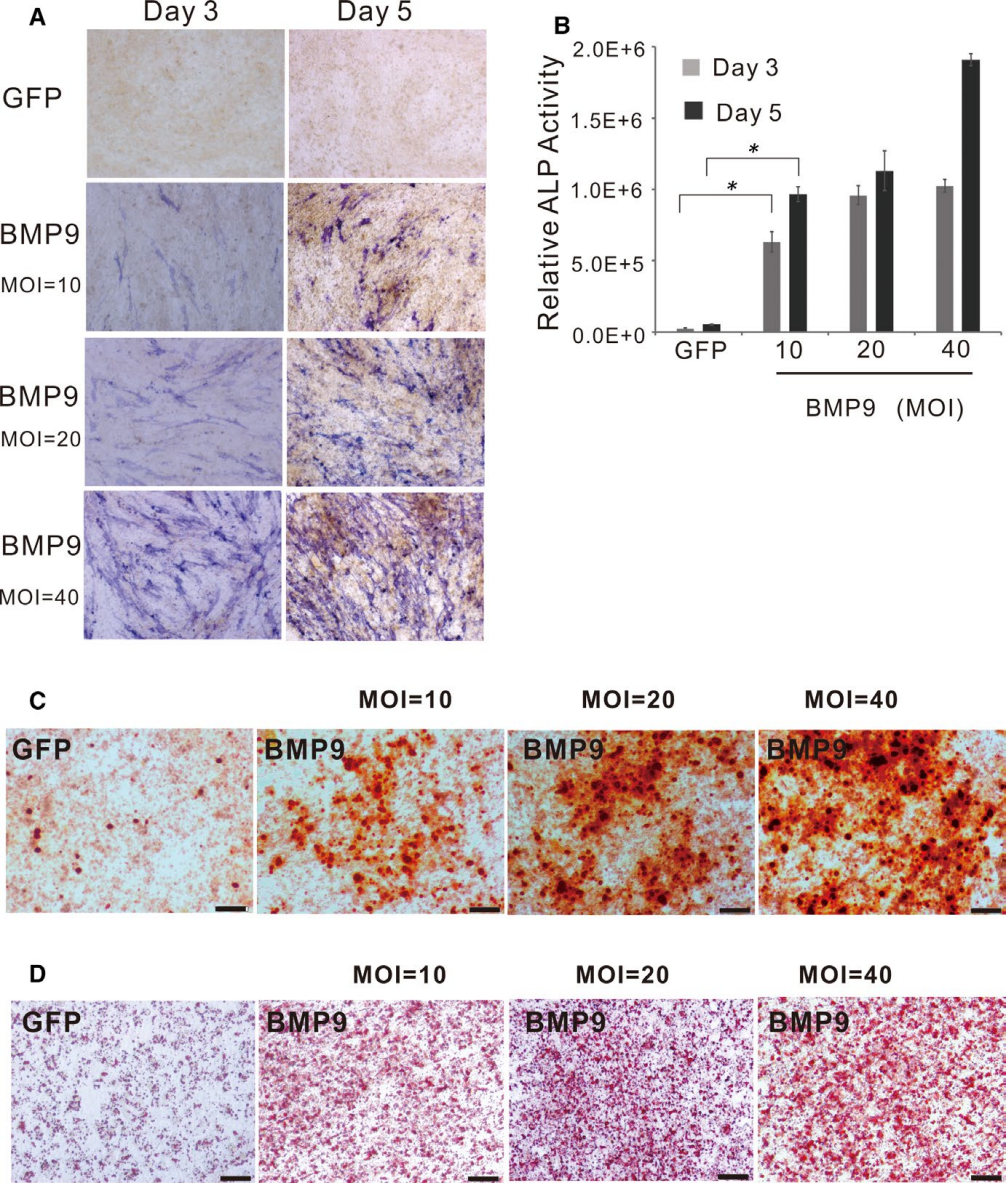
BMP9 induces expression of alkaline phosphatase (ALP), late‐stage mineralization and adipogenic differentiation in iTGMCs. A, ALP histochemical staining assay. B, Quantitative measurement of ALP activity. (*P < .05). C, Alizarin red staining demonstrated the late‐stage mineralization in BMP‐stimulated iTGMCs. D, Oil Red O staining demonstrated adipogenic differentiation in BMP‐stimulated iTGMCs

To test whether the iTGMCs have the osteogenic/odontogenic potential when induced by BMP9, we found that early osteogenic marker ALP was readily detected in BMP9 induced cells on as early as day 3, and that this effect was BMP9 dose‐dependent (Figure 3A). A similar dose‐dependent effect was found in the relative ALP activity in iTGMCs when induced by BMP9 (Figure 3B). Alizarin red staining further demonstrated the late‐stage mineralization in BMP‐stimulated iTGMCs (Figure 3C).

**FIGURE 4:**
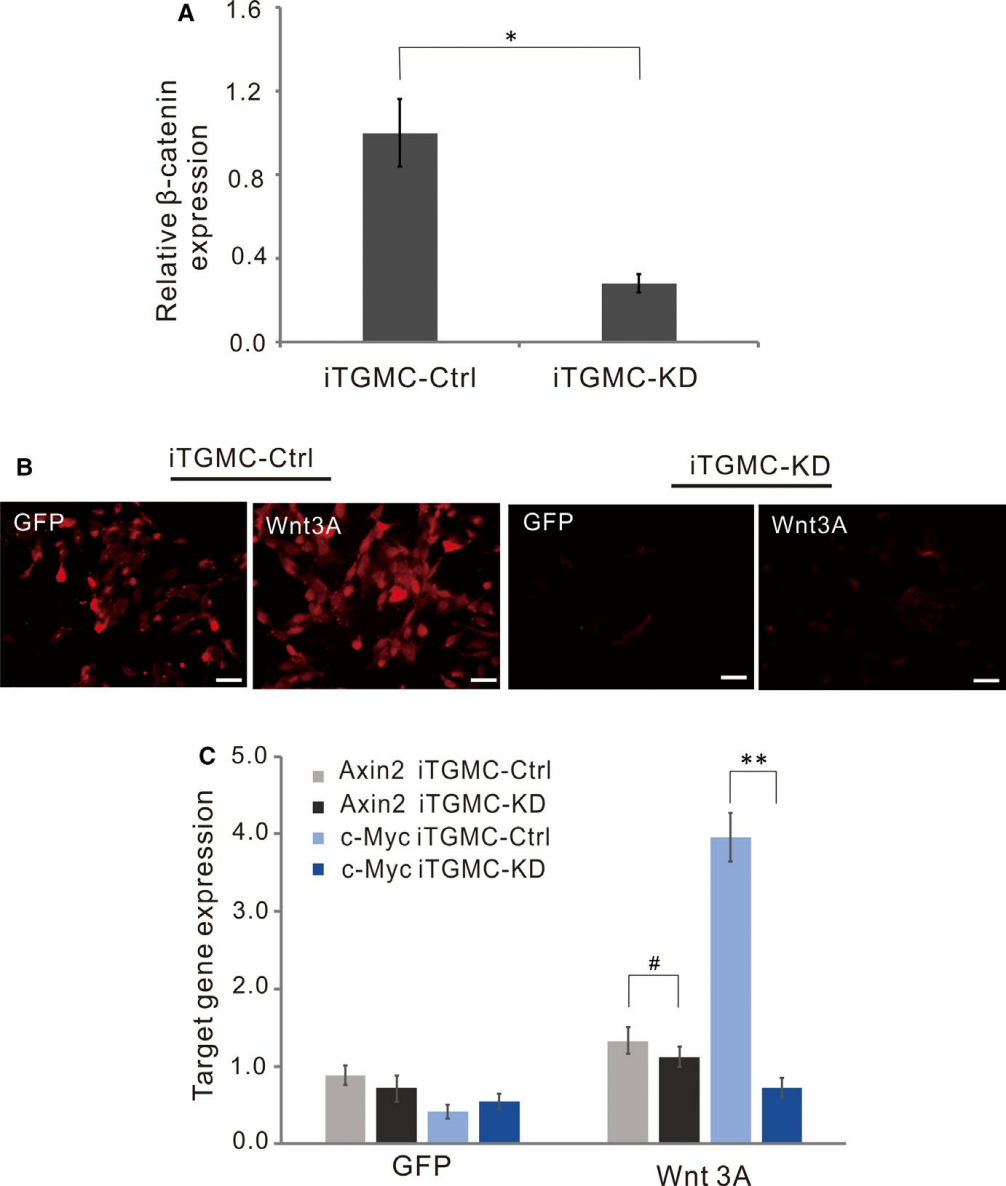
Knockdown of the expression of β‐catenin in iTGMCs by siRNAs targeting mouse β‐catenin. A, The knockdown efficiency of endogenous β‐catenin expression in iTGMCs was confirmed by RT‐PCR. (*P < .05). B, Wnt3a‐induced β‐catenin accumulation can be effectively blocked by pBSG361‐simBC, as shown by immunofluorescence staining. C, Wnt3A‐induced expressions of Wnt/β‐catenin target genes were remarkably repressed in iTGMC‐KD as shown by RT‐PCR. (**P < .01)

To further test whether the BMP9‐induced osteogenic/odontogenic activity of iTGMCs requires the participation of canonical Wnt signalling as we found in immortalized stem cells of dental apical papilla (iSCAPs),^12^ the endogenous β‐catenin expression was knockdown by the multiplex siRNAs targeting mouse β‐catenin. QPCR analysis showed that the knockdown efficiency was about 70% (Figure 4A). We found that cytoplasmic/nuclear accumulation of β‐catenin was significantly diminished in iTGMC‐KD cells transduced with Ad‐Wnt3A when compared with that in the iTGMC‐Ctrl cells (Figure 4B). Thus, these results indicate that the expression of β‐catenin was effectively repressed in the iTGMC‐KD cells.

We then analysed the effect of knocking down of β‐catenin expression on the downstream events of Wnt/β‐catenin signalling pathway in iTGMCs. Sub‐confluent iTGMC‐KD and iTGMC‐Ctrl cells were infected with Ad‐Wnt3A, or Ad‐GFP, and the expressions of Axin2 and c‐Myc, two well‐characterized Wnt/β‐catenin downstream target genes, were examined. Results showed that the significant expressions of Axin2 and c‐Myc were induced in iTGMC‐Ctrl cells by Wnt3A and were effectively inhibited in iTGMC‐KD cells by contrast (Figure 4C). These results suggest that multiplex siRNAs targeting β‐catenin expression in iTGMC‐KD may effectively repress the functional activities of canonical Wnt signalling.

**FIGURE 5:**
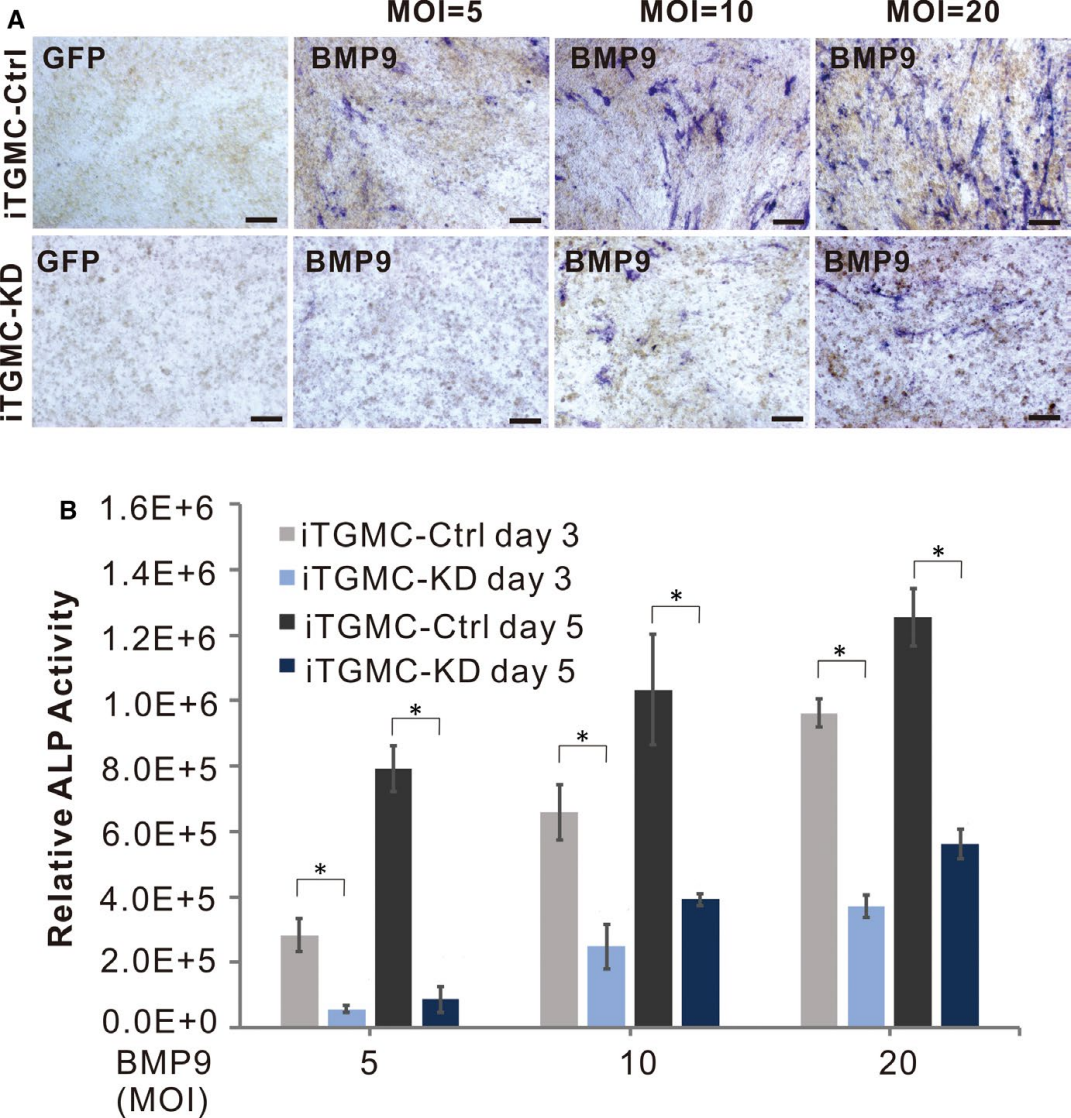
Silencing β‐catenin expression blunts BMP9‐induced expression of alkaline phosphatase (ALP) in iTGMCs. A, ALP histochemical staining assay showing the repressed expression of ALP by β‐catenin knockdown. B, Quantitative measurement of ALP activity. (*P < .05)

**FIGURE 6:**
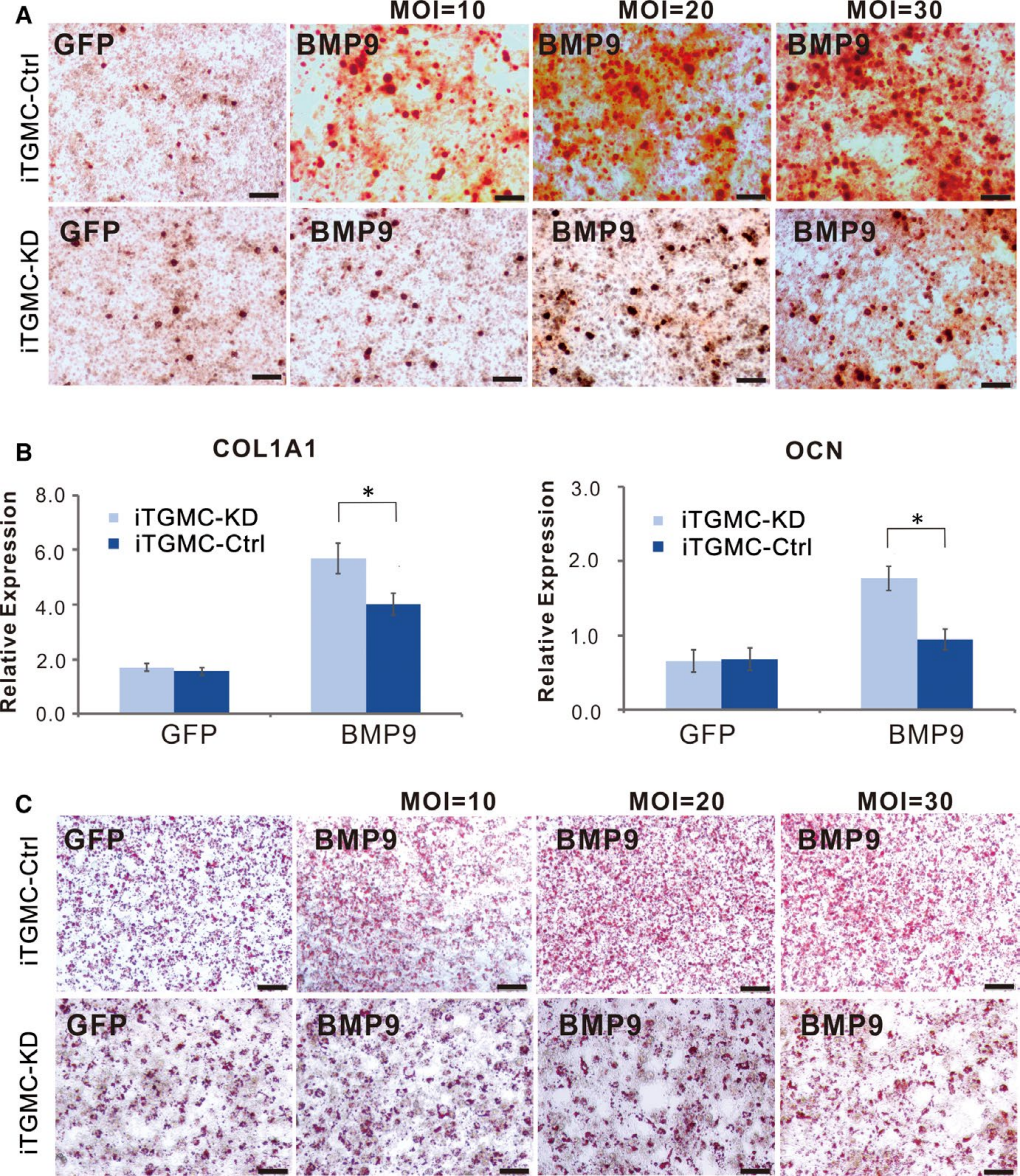
BMP9‐induced osteo/odontoblastic differentiation of iTGMCs requires participation of canonical Wnt signalling. A, Alizarin‐Red staining showed that Silencing β‐catenin expression significantly diminished matrix mineralization nodules formation induced by BMP9. B, Stronger adipogenic differentiation was found in iTGMC‐Ctrl than in iTGMC‐KD in a dose‐dependent fashion, shown by Oil Red O staining. C, Expression of bone specific markers induced by BMP9is significantly decreased in iTGMC‐KD group. (a) mouse collagen, type I, alpha 1 (Col1a1), (b) osteocalcin (OCN). Relative expression was calculated and GAPDH served as a reference gene. (*P < .05)

Additionally, when iTGMC‐Ctrl and iTGMC‐KD were stimulated by BMP9, we found that BMP9‐induced ALP activity staining was significantly weaker in iTGMC‐KD cells than in iTGMC‐Ctrl cells, although BMP9‐induced ALP staining was dose‐dependent in both cell lines (Figure 5A). Quantitative analysis of the relative ALP activity also demonstrated that BMP9‐induced ALP activities in iTGMC‐Ctrl were significantly stronger than that in iTGMC‐KD at each time point, and the effect was clearly dose‐dependent (Figure 5B). We further examined the late‐stage mineralization in BMP9‐stimulated iTGMCs. Results showed that Alizarin Red S staining was significantly weaker in iTMGC‐KD cells when compared with that in iTGMC‐Ctrl cells (Figure 6A). When the expression of COL1A1 (Collagen, type 1, alpha 1) and OCN (Osteocalcin) was examined, we found that Ad‐BMP9 significantly induced the expressions of COL1A1 and OCN in iTGMC‐Ctrl cells, which were evidently repressed in iTGMC‐KD cells (Figure 6B). These results indicate that BMP9 could effectively induce osteo/odontogenic activity of iTGMCs, and this process requires the participation of canonical Wnt signalling.

Furthermore, similar results as in mesenchymal stem cell (MSC)^10^, ^11^, ^19^, ^27^, ^35^ were found in iTGMCs. Oil Red O staining implied that BMP9 was capable of inducing adipogenic differentiation in iTGMCs (Figure 3D). Stronger adipogenic differentiation was found in iTGMC‐Ctrl than in iTGMC‐KD in a dose‐dependent fashion. These results suggest that this effect may also be affected by knocking down of β‐catenin expression (Figure 6C).

### 3.5 BMP9‐induced ectopic bone formation from iTGMCs can be attenuated by β‐catenin knockdown

**FIGURE 7:**
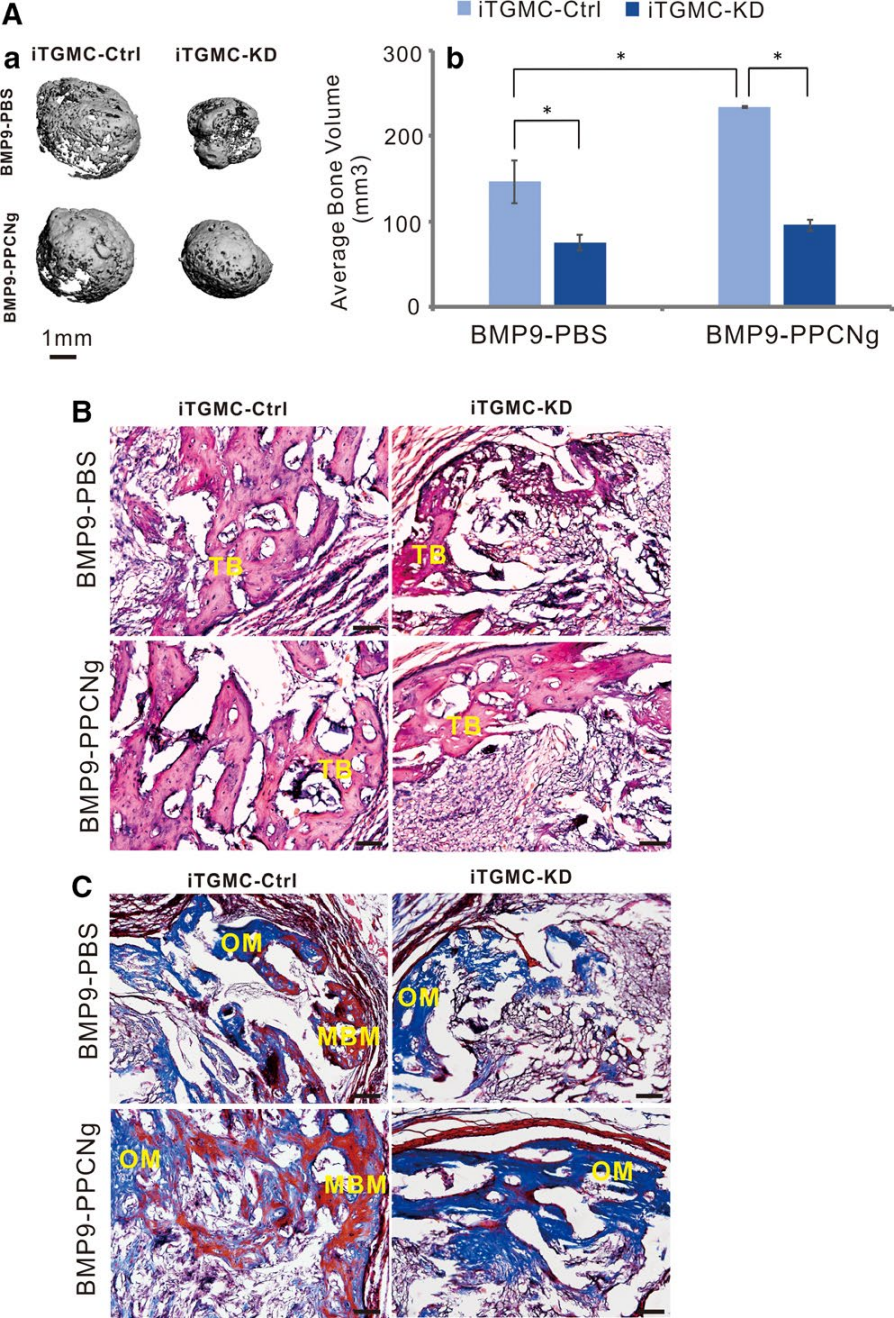
BMP9 induces ectopic bone formation from iTGMCs in vivo. A, BMP9 induced ectopic bone formation from iTGMCs in vivo was analysed by μCT. No masses were detectable in the Ad‐GFP‐infected iTGMC ‐KD and iTGMC‐Ctrl groups (a). The average bone volume was determined using the vivaCT 40, uCT V6.1 software(b). (*P < .05). B, Haematoxylin and eosin (H & E) staining showed that when transduced with BMP9 or in the presence of both BMP9 and PPCNg, trabecular bone formation was remarkably repressed in iTGMC‐KD compared with iTGMC‐Ctrl. C, Trichrome staining revealed that mature and mineralized bone matrices formation were also remarkably repressed in iTGMC‐KD cells compared with iTGMC‐Ctrl. MBM, mineralized bone matrix; TB, trabecular bone; OM, osteoid matrix. Bar = 50µM

We tested the in vivo effect of BMP9 on ectopic bone formation in iTGMCs via previously established stem cell implantation assay. Subconfluent iTGMC‐KD and iTGMC‐Ctrl cells were transduced with Ad‐BMP9 or Ad‐GFP, then the transduced cells were injected into the subcutaneous tissue of the flanks of athymic nude mice for 4 weeks. Robust bony masses were collected from BMP9 transduced iTGMC‐Ctrl, and much smaller masses were retrieved from the BMP9 transduced iTGMC‐KD groups, while no masses were recovered from the Ad‐GFP transduced groups. Micro‐CT imaging demonstrated more distinct dimension differences among these samples (Figure 7A‐a). Quantitative analysis of the average bone volumes found that significantly more robust bone formation was induced in BMP9 transduced iTGMC‐Ctrl cells than in BMP9 transduced iTGMC‐KD cells (Figure 7A‐b, *P* < .001), suggesting canonical Wnt signalling may be essential for BMP9‐induced bone formation in vivo. H & E histological evaluation further revealed that apparent trabecular bone was formed by iTGMC‐Ctrl cells when transduced with BMP9 or in the presence of both BMP9 and PPCNg. When the expression of β‐catenin was knockdown as in iTGMC‐KD cells, the trabecular bone formation would be significantly repressed (Figure 7B). Additionally, significant mature and mineralized bone matrices formed by iTGMC‐Ctrl cells transduced with BMP9, compared with significantly diminished bone maturation and mineralization in iTGMC‐KD cells transduced with BMP9, was confirmed by trichrome staining (Figure 7C). Taken together, these in vivo studies provide convincible evidences indicating that β‐catenin may have an important role in mediating BMP9‐induced osteo/odontoblastic differentiation of iTGMC cells.

## 4 DISCUSSION

Applying the TGMCs isolated from the tooth germ mesenchyme of mouse mandibular molar teeth, we demonstrated that these mesenchymal cells can be effectively immortalized with SV40 T antigen, and the immortalization is reversible. Furthermore, we found that iTGMCs retain osteogenic/odontogenic ability upon BMP9 stimulation, both in vitro and in vivo. To further investigated whether canonical Wnt/β‐catenin signalling has an essential role in BMP9 induced osteo/odontogenic signalling in iTGMCs as we previously reported in iSCAPs,^12^ a stable iTGMC‐KD line, in which β‐catenin expression was efficiently silenced, was established. We demonstrated that BMP9 induced ALP expression was significantly diminished in iTGMC‐KD by β‐catenin knockdown. Moreover, knocking down of β‐catenin expression led to a decrease in BMP9‐induced expression of osteocalcin, in vitro collagen synthesis and matrix mineralization, and adiopogenic differentiation of iTGMCs. Further in vivo studies provided convincible evidence revealing that BMP9‐transduced iTGMCs induced robust ectopic formation of bone tissue subcutaneously, which was significantly inhibited by knockdown of β‐catenin in iTGMCs. The results successfully demonstrated that canonical Wnt/β‐catenin signalling may have an important role in BMP9‐induced osteo/odontogenic signalling in iTGMCs.

Mineralized dentin and cementum tissues are formed in the late bell stage, respectively, by odontoblasts and cementoblasts differentiating from the dental mesenchyme. This process is regulated by multiple signalling pathways. For instance, multiple signalling molecules belonging to conserved signalling pathways such as BMP, Wnt, FGF and Hh families are expressed to guide dentin and cementum formation.^8^

Even though the search for leading transcriptional regulators of odontogenesis is far from over, the importance of Bmp family members has been recognized by many investigators. Several members in Bmp family have proven to be key participants in inducing terminal differentiation of odontoblast in vitro.^36^, ^37^, ^38^ Evidence also shows that Wnt signalling is associated with dentinogenesis. Loss of Wnt signalling would lead to compromised odontoblast maturation and reduced dentin formation, whereas over‐activation of Wnt signalling through constitutive stabilization of β‐catenin results in excessive dentin and cementum deposition.^39^, ^40^

BMP9 (aka, GDF2) is found by us as one of the least characterized yet the most potent osteogenic BMPs.^11^, ^35^ We have recently demonstrated that BMP9 is capable of efficiently inducing bone, cartilage and, to a lesser extent, adipocytes differentiation of iSCAPs.^13^ We also found that canonical Wnt signalling acted synergistically on BMP9‐induced osteo/odontoblastic differentiation of stem cells of dental apical papilla.^12^ It was reported that BMP9‐induced osteogenic differentiation in mesenchymal stem cell‐like C3H10T1/2 cells can be promoted by Wnt3a.^41^ In this study, we demonstrated that reversibly immortalized TGMCs retain osteogenic/odontogenic ability upon BMP9 stimulation, which also requires the participation of canonical Wnt signalling both in vitro and in vivo. These discoveries further suggest that the crosstalk between BMP9 and Wnt signalling may play an important role in BMP induced osteogenic/odontogenic differentiation in mesenchyme derived cells.

Acquired tooth loss resulted from trauma, dental caries and periodontal diseases presents a formidable challenge in controlling health care costs, in addition to its adverse effect on the quality of life. Although these problems can be managed successfully by conventional dental treatments through replacing the tooth with artificial materials or structures, such as fixed bridges, removable dentures, and dental implants, biotechnological developments based on biological findings are expected to bring further hope to restore the physiological functions of teeth.

While regeneration of fully functional teeth in living bodies has long been the dream of regenerative dentistry, one of the alternative approaches would be to engineer a tooth in vitro by seeding cells in tooth‐shaped scaffolds and implant it into patient's jaws.Stem cells derived from dental tissues or produced from somatic cells by iPS cell technology can serve as the seeding progenitors for tooth growth. Nonetheless, a number of difficult challenges has yet to be overcome. To produce odontogenic cells from somatic cells, they must be first reprogrammed to embryonic stem cells by iPS technology and programmed further to differentiate into dental epithelial or mesenchymal cell. However, at least for now, the molecular signatures of the epithelial and mesenchymal lineages that could be used in reprogramming is beyond the horizon of our knowledge. An alternative approach is to directly apply the stem cells discovered in adult teeth, namely the mesenchymal stem cells in dental papilla, pulp and periodontal ligament, which is limited mainly by availability and to a lesser extent by the complicated process of identification of stem cells. Aiming to address this problem, here we focus on the tooth germ mesenchymal cells, which may have been overlooked in regenerative dentistry. We demonstrated that iTGMCs can be expanded in long‐term culture, which exhibits at least two major advantages over iSCAPs or MSCs from other sources, including (a) TGMCs can be easily harvested and immortalized from developing third teeth in teenagers without causing damage to functional teeth; (b) stem cell identification may not be needed while the natural regenerative potential of iTGMCs being harnessed in regenerative dentistry.

## 5 CONCLUSIONS

In summary, we demonstrated that primary TGMCs can be reversibly immortalized by SV40 T antigen. These iTGMCs exhibit high proliferative activity and maintain long‐term cell proliferation under ex vivo culture conditions, which can be reversed by introducing FLP recombinase. We further demonstrated that the iTGMCs retained osteogenic/odontogenic ability upon BMP9 stimulation, but this process required the participation of canonical Wnt signalling both in vitro and in vivo. Therefore, BMP9 has a potential to be applied as an efficacious bio‐factor in osteo/odontogenic tissue engineering and tooth engineering. Furthermore, the iTGMCs may serve as an important seed cell resource for translational studies in tooth tissue engineering.

## CONFLICT OF INTEREST

The authors declare no competing conflicts of interests.

## AUTHOR CONTRIBUTION

**Wenping Luo:** Conceptualization (supporting); Data curation (equal); Funding acquisition (supporting); Methodology (equal); Writing‐original draft (supporting); Writing‐review & editing (supporting). **Linghuan Zhang:** Data curation (supporting); Investigation (supporting); Methodology (equal). **Bo Huang:** Conceptualization (supporting); Data curation (supporting); Investigation (supporting); Methodology (supporting). **HongMei Zhang:** Conceptualization (supporting); Methodology (supporting); Writing‐review & editing (supporting). **Yan Zhang:** Conceptualization (supporting); Investigation (supporting); Methodology (supporting). **FuGui Zhang:** Conceptualization (supporting); Investigation (supporting); Methodology (supporting). **PanPan Liang:** Data curation (supporting); Investigation (supporting); Methodology (supporting). **Qiuman Cheng:** Data curation (supporting); Investigation (supporting); Methodology (supporting). **Qianyu Cheng:** Investigation (supporting); Methodology (supporting). **Dongmei Tan:** Data curation (supporting); Investigation (supporting). **Yi Tan:** Data curation (supporting); Investigation (supporting); Methodology (supporting). **Jinlin Song:** Conceptualization (supporting); Data curation (supporting); Writing‐review & editing (supporting). **Tianyu Zhao:** Conceptualization (supporting); Data curation (supporting); Funding acquisition (supporting); Methodology (supporting). **Rex C Haydon:** Investigation (supporting); Methodology (supporting); Writing‐review & editing (supporting). **Russell R Reid:** Investigation (supporting); Methodology (supporting); Writing‐review & editing (supporting). **Hue H Luu:** Investigation (supporting); Methodology (supporting); Writing‐review & editing (supporting). **Michael J Lee:** Investigation (supporting); Methodology (supporting); Writing‐review & editing (supporting). **Mostafa El Dafrawy:** Methodology (supporting); Writing‐review & editing (supporting). **Ping Ji:** Conceptualization (supporting); Data curation (supporting); Funding acquisition (supporting); Methodology (supporting); Writing‐review & editing (supporting). **Tong‐Chuan He:** Conceptualization (equal); Funding acquisition (equal); Methodology (equal); Project administration (equal); Writing‐original draft (equal); Writing‐review & editing (equal). **Liming Gou:** Conceptualization (equal); Funding acquisition (equal); Methodology (equal); Project administration (equal); Writing‐original draft (equal); Writing‐review & editing (equal).

## Data Availability

The data that supporting the findings of this study are available from the corresponding authors upon request.

## References

[1] Tucker A , Sharpe P . The cutting‐edge of mammalian development; how the embryo makes teeth. Nat Rev Genet. 2004;5(7):499‐508. 15211352 10.1038/nrg1380

[2] Fukumoto S , Yamada Y . Review: extracellular matrix regulates tooth morphogenesis. Connect Tissue Res. 2005;46(4–5):220‐226. 16546825 10.1080/03008200500344017

[3] Kollar EJ , Baird GR . The influence of the dental papilla on the development of tooth shape in embryonic mouse tooth germs. J Embryol Exp Morphol. 1969;21(1):131‐148. 5765787

[4] Kollar EJ , Baird GR . Tissue interactions in embryonic mouse tooth germs. II. The inductive role of the dental papilla. J Embryol Exp Morphol. 1970;24(1):173‐186. 5487155

[5] Mina M , Kollar EJ . The induction of odontogenesis in non‐dental mesenchyme combined with early murine mandibular arch epithelium. Arch Oral Biol. 1987;32(2):123‐127. 3478009 10.1016/0003-9969(87)90055-0

[6] Ono M , Oshima M , Ogawa M , et al. Practical whole‐tooth restoration utilizing autologous bioengineered tooth germ transplantation in a postnatal canine model. Sci Rep. 2017;7:44522. 28300208 10.1038/srep44522 PMC5353657

[7] Goldberg M . Pulp healing and regeneration: more questions than answers. Adv Dent Res. 2011;23(3):270‐274. 21677077 10.1177/0022034511405385

[8] Jussila M , Thesleff I . Signaling networks regulating tooth organogenesis and regeneration, and the specification of dental mesenchymal and epithelial cell lineages. Cold Spring Harbor Perspect Biol. 2012;4(4):A008425. 10.1101/cshperspect.a008425 PMC3312678 22415375

[9] Balic A , Thesleff I . Tissue interactions regulating tooth development and renewal. Curr Top Dev Biol. 2015;115:157‐186. 26589925 10.1016/bs.ctdb.2015.07.006

[10] Kang Q , Song WX , Luo Q , et al. A comprehensive analysis of the dual roles of BMPs in regulating adipogenic and osteogenic differentiation of mesenchymal progenitor cells. Stem Cells Dev. 2009;18(4):545‐559. 18616389 10.1089/scd.2008.0130 PMC3132950

[11] Luther G , Wagner ER , Zhu G , et al. BMP‐9 induced osteogenic differentiation of mesenchymal stem cells: molecular mechanism and therapeutic potential. Curr Gene Ther. 2011;11(3):229‐240. 21453282 10.2174/156652311795684777

[12] Zhang H , Wang J , Deng F , et al. Canonical Wnt signaling acts synergistically on BMP9‐induced osteo/odontoblastic differentiation of stem cells of dental apical papilla (scaps). Biomaterials. 2015;39:145‐154. 25468367 10.1016/j.biomaterials.2014.11.007 PMC4258144

[13] Wang J , Zhang H , Zhang W , et al. Bone morphogenetic protein‐9 effectively induces osteo/odontoblastic differentiation of the reversibly immortalized stem cells of dental apical papilla. Stem Cells Dev. 2014;23(12):1405‐1416. 24517722 10.1089/scd.2013.0580 PMC4046201

[14] Wu N , Zhang H , Deng F , et al. Overexpression of Ad5 precursor terminal protein accelerates recombinant adenovirus packaging and amplification in HEK‐293 packaging cells. Gene Ther. 2014;21(7):629‐637. 24784448 10.1038/gt.2014.40

[15] Westerman KA , Leboulch P . Reversible immortalization of mammalian cells mediated by retroviral transfer and site‐specific recombination. Proc Natl Acad Sci USA. 1996;93(17):8971‐8976. 8799138 10.1073/pnas.93.17.8971 PMC38579

[16] Li M , Chen Y , Bi Y , et al. Establishment and characterization of the reversibly immortalized mouse fetal heart progenitors. Int J Med Sci. 2013;10(8):1035‐1046. 23801891 10.7150/ijms.6639 PMC3691803

[17] Yang K , Chen J , Jiang W , et al. Conditional immortalization establishes a repertoire of mouse melanocyte progenitors with distinct melanogenic differentiation potential. J Invest Dermatol. 2012;132(10):2479‐2483. 22592154 10.1038/jid.2012.145 PMC4083699

[18] Yu X , Chen L , Wu K , et al. Establishment and functional characterization of the reversibly immortalized mouse glomerular podocytes (imPODs). Genes Dis. 2018;5(2):137‐149. 30258943 10.1016/j.gendis.2018.04.003 PMC6147083

[19] Kang Q , Sun MH , Cheng H , et al. Characterization of the distinct orthotopic bone‐forming activity of 14 BMPs using recombinant adenovirus‐mediated gene delivery. Gene Ther. 2004;11(17):1312‐1320. 15269709 10.1038/sj.gt.3302298

[20] Zhao C , Wu N , Deng F , et al. Adenovirus‐mediated gene transfer in mesenchymal stem cells can be significantly enhanced by the cationic polymer polybrene. PLoS One. 2014;9(3):E92908. 24658746 10.1371/journal.pone.0092908 PMC3962475

[21] Wang X , Yuan C , Huang B , et al. Developing a versatile shotgun cloning strategy for single‐vector‐based multiplex expression of short interfering RNAs (siRNAs) in mammalian cells. ACS Synth Biol. 2019;8(9):2092‐2105. 31465214 10.1021/acssynbio.9b00203 PMC6760290

[22] Zhang W , Deng ZL , Chen L , et al. Retinoic acids potentiate BMP9‐induced osteogenic differentiation of mesenchymal progenitor cells. PLoS One. 2010;5(7):E11917. 20689834 10.1371/journal.pone.0011917 PMC2912873

[23] Lamplot JD , Liu B , Yin L , et al. Reversibly immortalized mouse articular chondrocytes acquire long‐term proliferative capability while retaining chondrogenic phenotype. Cell Transplant. 2015;24(6):1053‐1066. 24800751 10.3727/096368914X681054

[24] Si W , Kang Q , Luu HH , et al. CCN1/Cyr61 Is regulated by the canonical Wnt signal and plays an important role in Wnt3A‐induced osteoblast differentiation of mesenchymal stem cells. Mol Cell Biol. 2006;26(8):2955‐2964. 16581771 10.1128/MCB.26.8.2955-2964.2006 PMC1446962

[25] Peng Y , Kang Q , Cheng H , et al. Transcriptional characterization of bone morphogenetic proteins (BMPs)‐mediated osteogenic signaling. J Cell Biochem. 2003;90(6):1149‐1165. 14635189 10.1002/jcb.10744

[26] Zhu GH , Huang J , Bi Y , et al. Activation of RXR and RAR signaling promotes myogenic differentiation of myoblastic C2C12 cells. Differentiation. 2009;78(4):195‐204. 19560855 10.1016/j.diff.2009.06.001 PMC2829657

[27] Cheng H , Jiang W , Phillips FM , et al. Osteogenic activity of the fourteen types of human bone morphogenetic proteins (BMPs). J Bone Joint Surg Am Vol. 2003;85(8):1544‐1552. 10.2106/00004623-200308000-00017 12925636

[28] Sharff KA , Song WX , Luo X , et al. Hey1 basic helix‐loop‐helix protein plays an important role in mediating BMP9‐induced osteogenic differentiation of mesenchymal progenitor cells. J Biol Chem. 2009;284(1):649‐659. 18986983 10.1074/jbc.M806389200 PMC2610517

[29] Ye J , Wang J , Zhu Y , et al. A thermoresponsive polydiolcitrate‐gelatin scaffold and delivery system mediates effective bone formation from BMP9‐transduced mesenchymal stem cells. Biomed Mater. 2016;11(2):025021‐025021. 27097687 10.1088/1748-6041/11/2/025021

[30] Luo X , Chen J , Song WX , et al. Osteogenic BMPs promote tumor growth of human osteosarcomas that harbor differentiation defects. Lab Invest. 2008;88(12):1264‐1277. 18838962 10.1038/labinvest.2008.98 PMC9901484

[31] Liao Z , Nan G , Yan Z , et al. The anthelmintic drug niclosamide inhibits the proliferative activity of human osteosarcoma cells by targeting multiple signal pathways. Curr Cancer Drug Targets. 2015;15(8):726‐738. 26118906 10.2174/1568009615666150629132157

[32] Li R , Zhang W , Cui J , et al. Targeting BMP9‐promoted human osteosarcoma growth by inactivation of notch signaling. Curr Cancer Drug Targets. 2014;14(3):274‐285. 24605944 10.2174/1568009614666140305105805

[33] Lu S , Wang J , Ye J , et al. Bone morphogenetic protein 9 (BMP9) induces effective bone formation from reversibly immortalized multipotent adipose‐derived (IMAD) mesenchymal stem cells. Am J Transl Res. 2016;8(9):3710‐3730. 27725853 PMC5040671

[34] Wang N , Zhang W , Cui J , et al. The piggybac transposon‐mediated expression of SV40 T antigen efficiently immortalizes mouse embryonic fibroblasts (MEFS). PLoS One. 2014;9(5):E97316. 24845466 10.1371/journal.pone.0097316 PMC4028212

[35] Luu HH , Song WX , Luo X , et al. Distinct roles of bone morphogenetic proteins in osteogenic differentiation of mesenchymal stem cells. J Orthop Res. 2007;25(5):665‐677. 17290432 10.1002/jor.20359

[36] Nakashima M . Induction of dentin formation on canine amputated pulp by recombinant human bone morphogenetic proteins (BMP)‐2 and ‐4. J Dent Res. 1994;73(9):1515‐1522. 7929986 10.1177/00220345940730090601

[37] Taşlı PN , Aydın S , Yalvaç ME , et al. BMP 2 and BMP 7 induce odonto‐ and osteogenesis of human tooth germ stem cells. Appl Biochem Biotechnol. 2014;172(6):3016‐3025. 24477555 10.1007/s12010-013-0706-0

[38] Zhu L , Ma J , Mu R , et al. Bone morphogenetic protein 7 promotes odontogenic differentiation of dental pulp stem cells in vitro. Life Sci. 2018;202:175‐181. 29555587 10.1016/j.lfs.2018.03.026

[39] Bae CH , Kim TH , Ko SO , et al. Wntless regulates dentin apposition and root elongation in the mandibular molar. J Dent Res. 2015;94(3):439‐445. 25595365 10.1177/0022034514567198 PMC4814015

[40] Kim TH , Lee JY , Baek JA , et al. Constitutive stabilization Of ß‐catenin in the dental mesenchyme leads to excessive dentin and cementum formation. Biochem Biophys Res Commun. 2011;412(4):549‐555. 21854758 10.1016/j.bbrc.2011.07.116

[41] Zhang X , Lin L‐B , Xu D‐J , et al. Wnt3a enhances bone morphogenetic protein 9‐induced osteogenic differentiation of C3H10T1/2 cells. Chin Med J. 2013;126(24):4758‐4763. 24342325

