# Corrigendum

**DOI:** 10.1111/jcmm.17130

**Published:** 2022-05-07

In Wenping Luo et al.,^1^ there are several errors in the figure legends of Figure 2 and Figure 6, the figure panel labelling in Figure 1 and Figure 6B, and an image assembly error in Figure 7B. The correct legends and figures are shown below. The authors confirm that all results and conclusions of this article remain unchanged.

**FIGURE 1:**
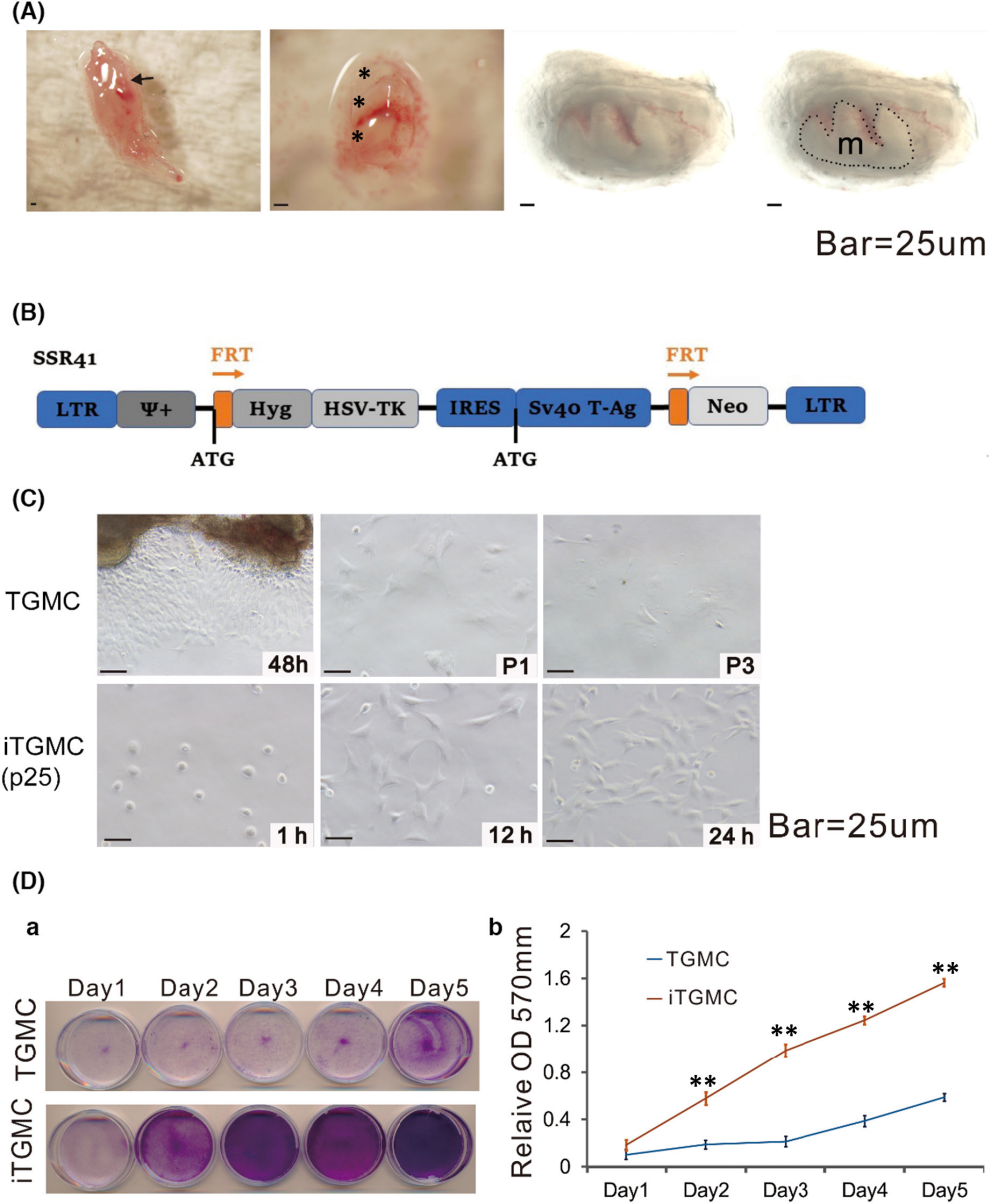
Isolation of mouse tooth germ mesenchyme cells (TGMCs) from mandibular late bell stage molar tooth germs and reversible immortalization of TGMCs (iTGMCs). (A) Isolation of late bell stage molar tooth germs from mandible on postnatal day 0. (short arrow, mandibular molar tooth germs attached to the mandible. *dissected mandibular molar tooth germs. m, tooth germ mesenchyme (Captured under transmission light)). (B) Schematic representation of immortalization vector SSR#41, which contains the hygromycin and SV40 T antigen expression cassette flanked with FRT sites and can be removed by the Flippase (FLP) recombinase. (C) Morphology of the primary TGMCs and iTGMCs. Bar =25 µM. (a)TGMCs were harvested from digested tooth germs mesenchyme after 48 h culture and maintained up to three passages(P3). (b) iTGMCs were seeded at a low cell density and photographed at the indicated time point. iTGMCs were readily maintained indefinitely (passage 25 is shown). (D) Cell proliferation assessed by crystal violet staining. (a) TGMCs (P2) and iTGMCs were seeded at 30 mm dish with the same initial density and then fixed with paraformaldehyde for crystal violet staining after cultured for the indicated time scale. (b) The stained cells were dissolved in 10% acetic acid and optical absorbance was measured at 570 to 590 nm. (**p < 0.001)

**FIGURE 2:**
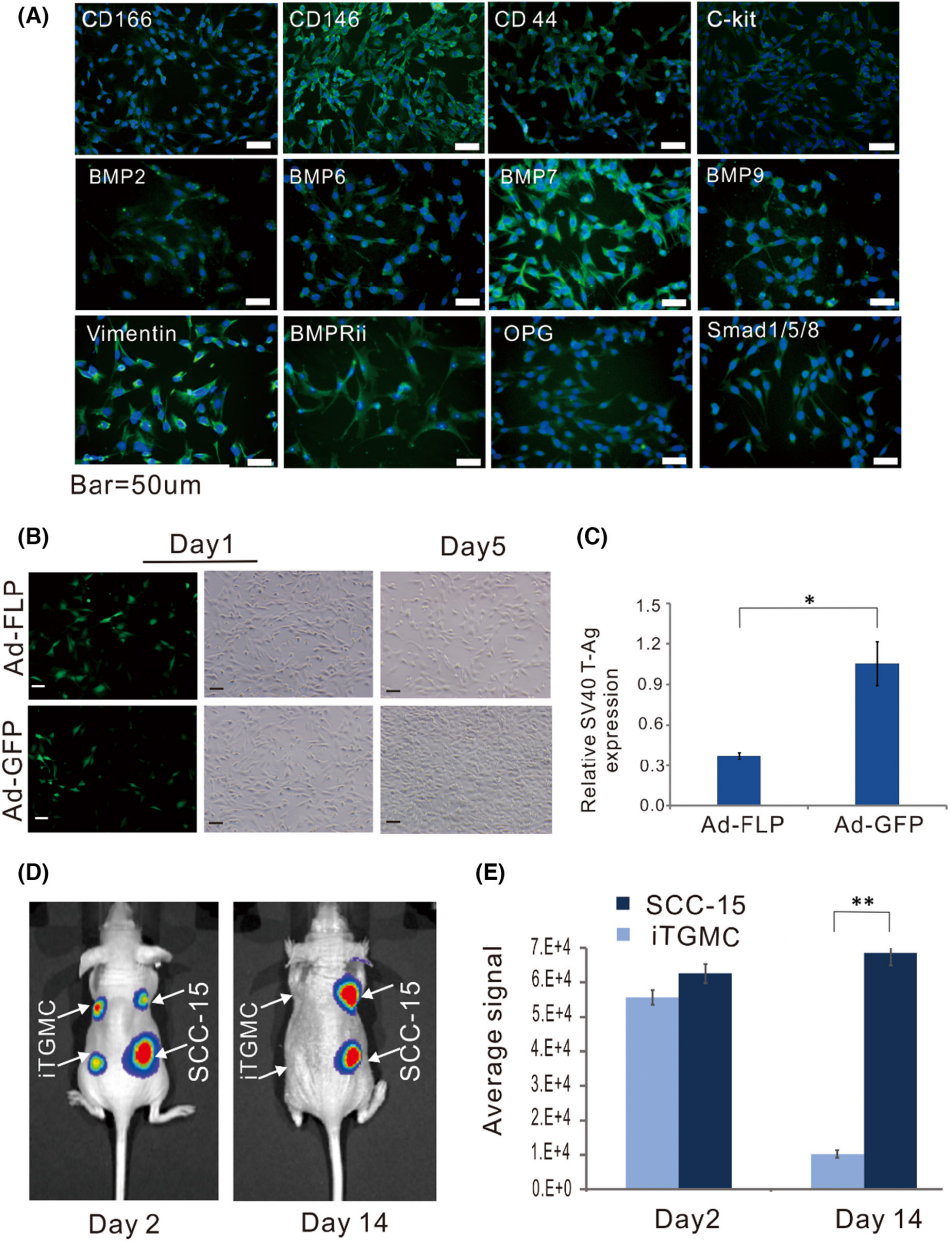
Characterization of Reversibly immortalized TGMCs (iTGMCs). (A) Expression of mesenchymal and/or progenitor markers in iTGMCs. IgG and minus primary antibody staining were performed as negative controls (not shown). (B) The proliferative activity of iTGMCs decreases after removal of SV40 T antigen via FLP recombinase. (C) Removal of SV40 T antigen in iTGMCs confirmed by RT‐qPCR analysis 3 days after infection. (*p < 0.05). (D) Analysis of the tumorigenic risk in vivo. SCC‐15 and iTGMCs stably expressing the firefly luciferase were injected into the athymic nude mice subcutaneously. Compared with the SCC‐15 group, no masses were detectable in the iTGMCs group at Day 14. (E) The average bioluminescence signals were quantitatively analysed by the Living Image software. (**p < 0.01). Representative results are presented

**FIGURE 6:**
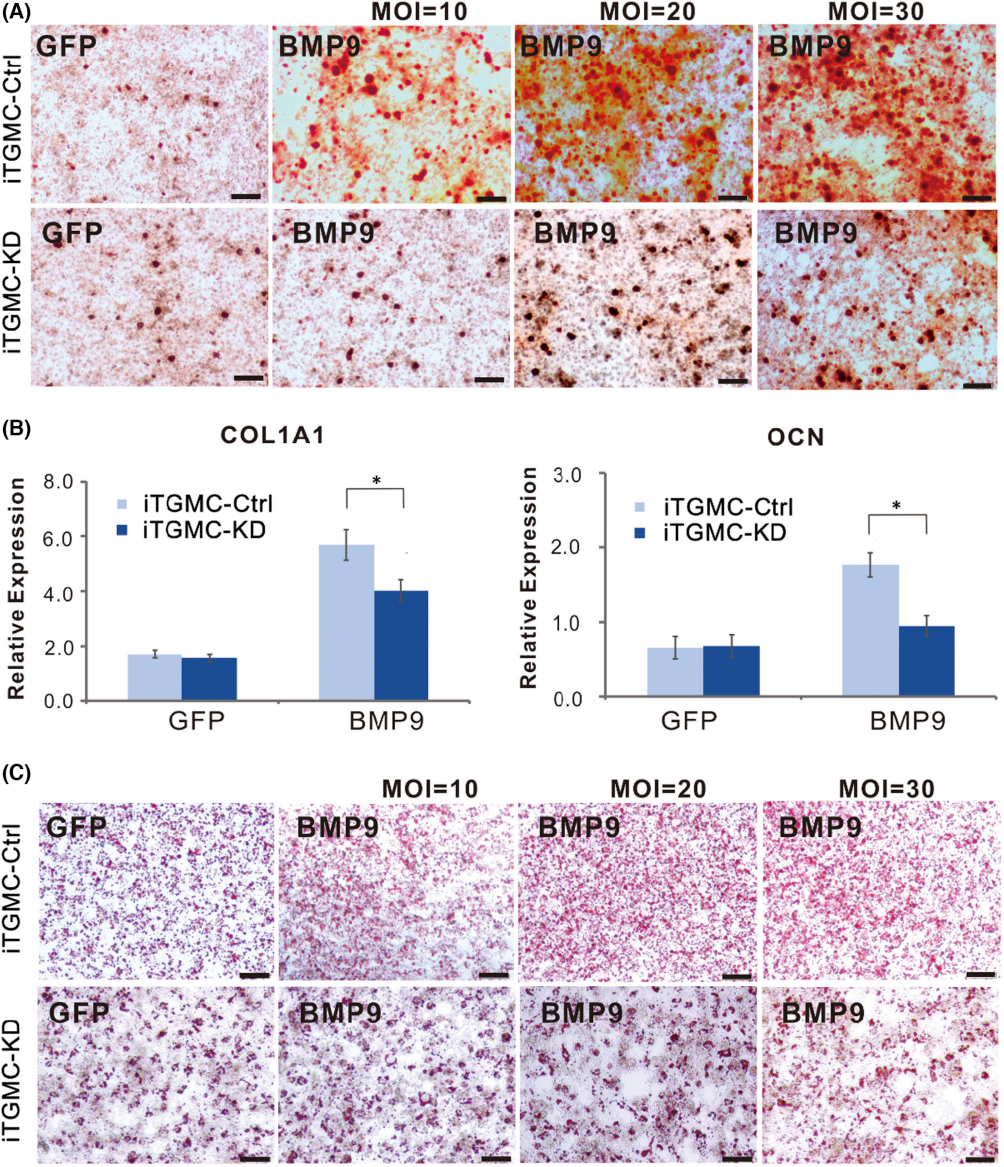
BMP9‐induced osteo/odontoblastic differentiation of iTGMCs requires participation of canonical Wnt signalling. (A) Alizarin Red staining showed that Silencing β‐catenin expression significantly diminished matrix mineralization nodules formation induced by BMP9. (B) Expression of bone specific markers induced by BMP9is significantly decreased in iTGMC‐KD group. (a) mouse collagen, type I, alpha 1 (Col1a1), (b) osteocalcin (OCN). Relative expression was calculated and GAPDH served as a reference gene. (*p < 0.05). (C) Stronger adipogenic differentiation was found in iTGMC‐Ctrl than in iTGMC‐KD in a dose‐dependent fashion, shown by Oil Red O staining

**FIGURE 7:**
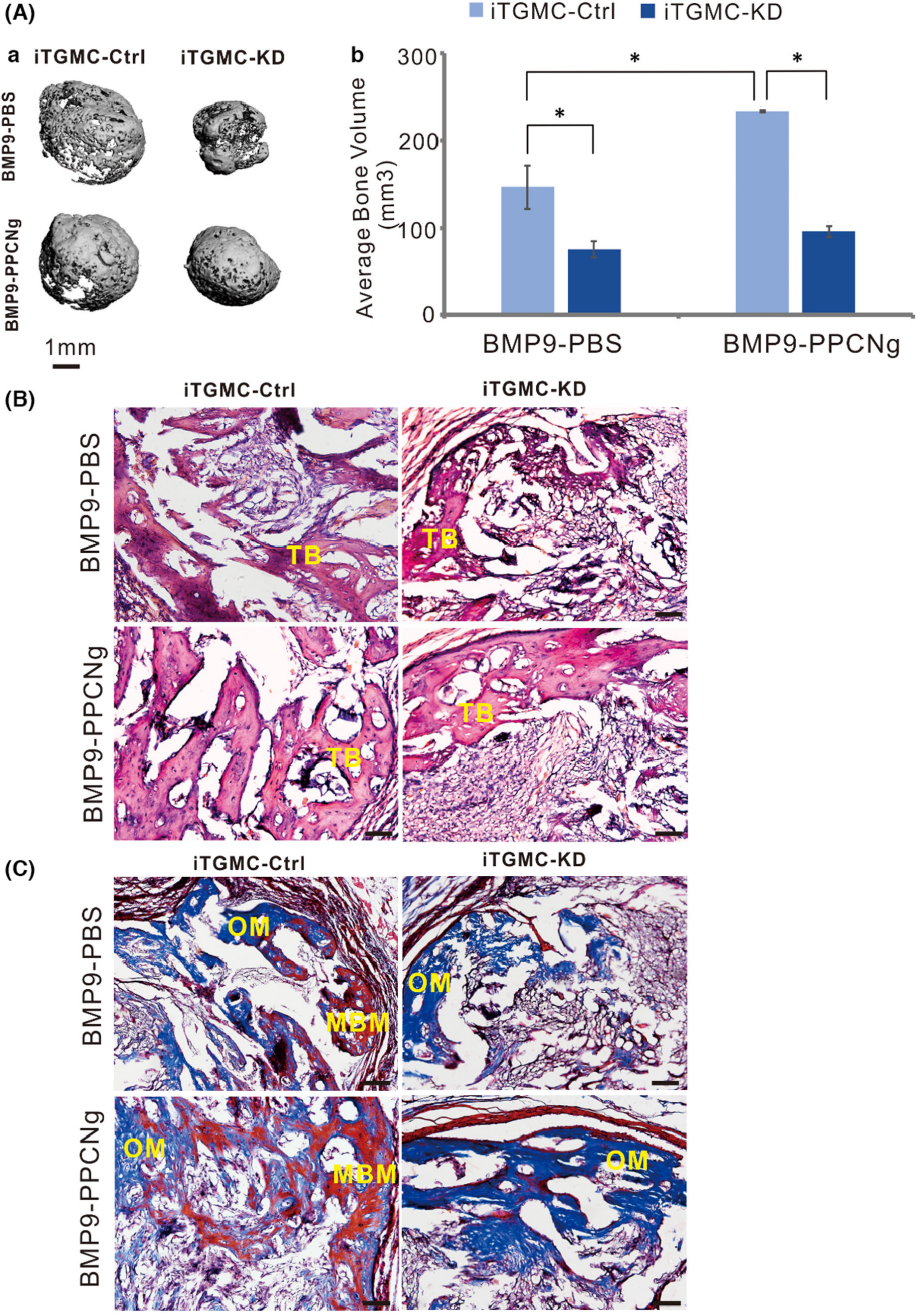
BMP9 induces ectopic bone formation from iTGMCs in vivo. (A) BMP9‐induced ectopic bone formation from iTGMCs in vivo was analysed by μCT. No masses were detectable in the Ad‐GFP‐infected iTGMC ‐KD and iTGMC‐Ctrl groups (a). The average bone volume was determined using the vivaCT 40, uCT V6.1 software(b). (*p < 0.05). (B) Haematoxylin and eosin (H & E) staining showed that when transduced with BMP9 or in the presence of both BMP9 and PPCNg, trabecular bone formation was remarkably repressed in iTGMC‐KD compared with iTGMC‐Ctrl. (C) Trichrome staining revealed that mature and mineralized bone matrices formation were also remarkably repressed in iTGMC‐KD cells compared with iTGMC‐Ctrl. MBM, mineralized bone matrix; TB, trabecular bone; OM, osteoid matrix. Bar =50 µM
